# CBCT in Dental Research: Is Complexity Necessary? An Exploratory Proof-of-Concept Study

**DOI:** 10.3390/jfb17080385

**Published:** 2026-08-04

**Authors:** Selma Tekin, Karim Oumalou, Selim Tekin, Rui B. Ruben, Margarida Franco, Nuno Alves, Cláudia Barbosa, Sandra Gavinha, Maria Conceição Manso, Tiago Reis

**Affiliations:** 1 Faculty of Health Sciences, University Fernando Pessoa, 4200-150 Porto, Portugal; drtekinselma@gmail.com (S.T.); karim.oumalou@gmail.com (K.O.); 2 Private Practice, 33600 Pessac, France; drtekinselim@gmail.com; 3 CDRSP, Polytechnic University of Leiria, 2430-028 Marinha Grande, Portugal; rui.ruben@ipleiria.pt (R.B.R.); margarida.franco@ipleiria.pt (M.F.); nuno.alves@ipleiria.pt (N.A.); 4 FP-I3ID, FP-BHS, Health Sciences Faculty, University Fernando Pessoa, 4249-004 Porto, Portugal; cbarbosa@ufp.edu.pt (C.B.); sgavinha@ufp.edu.pt (S.G.); 5 RISE-Health, Faculty of Health Sciences, University Fernando Pessoa, Fernando Pessoa Teaching and Culture Foundation, 4200-150 Porto, Portugal; 6 LAQV-REQUIMTE, University of Porto, 4051-401 Porto, Portugal

**Keywords:** 3D printing, cone beam computed tomography, dental education, dental research, dentistry

## Abstract

This exploratory proof-of-concept study aimed to develop and preliminarily evaluate a 3D-printed holder designed for reuse in cone beam computed tomography (CBCT) in dental research, particularly for comparative studies requiring pre- and post-intervention tooth assessment. The holder was designed using computer-aided design software and fabricated from polylactic acid using 3D printing. A conventional alginate-based holder was used for comparison. The comparison therefore concerned two complete positioning systems with different geometries and modes of adaptation to the CBCT headrest. The two positioning devices were independently placed by two operators with different levels of experience, and all CBCT scans were acquired by the same operator. The procedure was repeated after a 5-day interval. Root canal morphology was assessed using volumetric and surface measurements, as well as voxel counts. The independently segmented canal models were qualitatively displayed together in their native coordinate space to visualize positional consistency between acquisitions. Paired analyses showed substantially lower positional deviations with the 3D-printed holder than with the conventional holder in all four acquisition comparisons (Holm-adjusted *p* < 0.001). Mean deviations ranged from 0.126 to 0.172 mm for the 3D-printed holder and from 3.626 to 8.318 mm for the conventional holder. Morphometric parameters derived from 3D analysis remained identical across all acquisitions, regardless of operator, time point, or positioning method. Qualitative 3D analysis showed near-complete overlap for the 3D-printed holder and clear spatial discrepancies for the conventional holder. Under the evaluated conditions, the 3D-printed holder exhibited less positional variability than the conventional alginate-based holder.

## 1. Introduction

Developing, maintaining, and sustaining undergraduate research initiatives benefit academic institutions, faculty mentors, and students. By integrating research into academic courses, students can enhance their critical thinking. Those who participate in research projects develop greater intellectual autonomy, stronger intrinsic motivation to learn, and a more active role in their education. Consequently, the research experience has a profoundly positive impact on their professional development as they prepare for their careers [1,2]. The literature highlights several barriers that undergraduate students face in conducting research, namely, a lack of time to engage in research, insufficient laboratory facilities and a lack of funding [2].

Micro-computed tomography (Micro-CT) is considered a reference method for high-resolution three-dimensional analysis of external and internal dental anatomy. However, its high cost and lengthy acquisition and reconstruction procedures may limit its accessibility, particularly in undergraduate research settings [3,4]. Cone beam computed tomography (CBCT) provides three-dimensional imaging with shorter acquisition times, broader availability, and the possibility of scanning multiple teeth simultaneously [3,4]. Depending on the acquisition protocol and equipment, CBCT can provide submillimetric spatial resolution suitable for several in vitro dental applications [5]. Datasets can be exported in Digital Imaging and Communications in Medicine (DICOM) format and processed using third-party software for segmentation, morphometric analysis, and three-dimensional reconstruction [4,5,6]. These characteristics make CBCT a practical alternative for selected dental research applications in which Micro-CT is unavailable or unnecessary.

CBCT has been used in in vitro studies across several dental fields, including endodontics [7,8,9,10,11,12,13,14,15], conservative dentistry [16,17,18], prosthodontics [19,20], and dental anatomy [21,22,23,24]. In comparative or longitudinal experimental designs, reliable interpretation depends not only on standardized acquisition parameters but also on reproducible specimen positioning. Variations in tooth orientation or holder placement between acquisitions may alter the spatial relationship of the structures under evaluation, complicate image comparison, and increase reliance on post-processing registration procedures. Positioning variability may therefore constitute an additional source of methodological error, particularly in studies requiring pre- and post-intervention assessment of the same specimens.

Despite its methodological relevance, positioning devices are not consistently described across in vitro CBCT studies and are frequently single-use or study-specific. Differences in holder geometry, fixation material, adaptation to the CBCT headrest, and specimen repositioning procedures may limit reproducibility within and between investigations. A standardized modular device designed for reuse could reduce the need to manufacture multiple supports, simplify repeated positioning, and improve methodological consistency.

These holders are typically single-use, case-specific, and not designed for reuse. To the best of the authors’ knowledge, no previous study has evaluated a modular 3D-printed holder specifically designed for reuse and repeated tooth positioning during CBCT acquisition. We hypothesized that the 3D-printed holder designed for reuse would produce lower positioning variability than the conventional alginate-based holder under the evaluated experimental conditions. Therefore, this exploratory proof-of-concept study aimed to develop and preliminarily evaluate the proposed device by comparing its positioning repeatability with that of a conventional alginate-based holder.

## 2. Materials and Methods

The study was conducted in accordance with the Declaration of Helsinki and approved by the ethics committee of Fernando Pessoa University (FCS/MMED 504/23-20; 1 February 2024). Written informed consent for the use of the extracted teeth for research purposes was obtained from all donors.

A prototype of the 3D-printed holder was initially constructed manually to determine its dimensions and evaluate its stability on the CBCT system (Carestream CS 8200 3D^®^, Carestream Dental LLC, Atlanta, GA, USA). The prototype was shaped using a rubber container (Mestra^®^, MESTRA, Biscaye, Spain) as a reference model and fabricated with self-curing acrylic resin (Methax Ortho^®^, MakeVale Ltd., London, UK).

**Figure 1:**
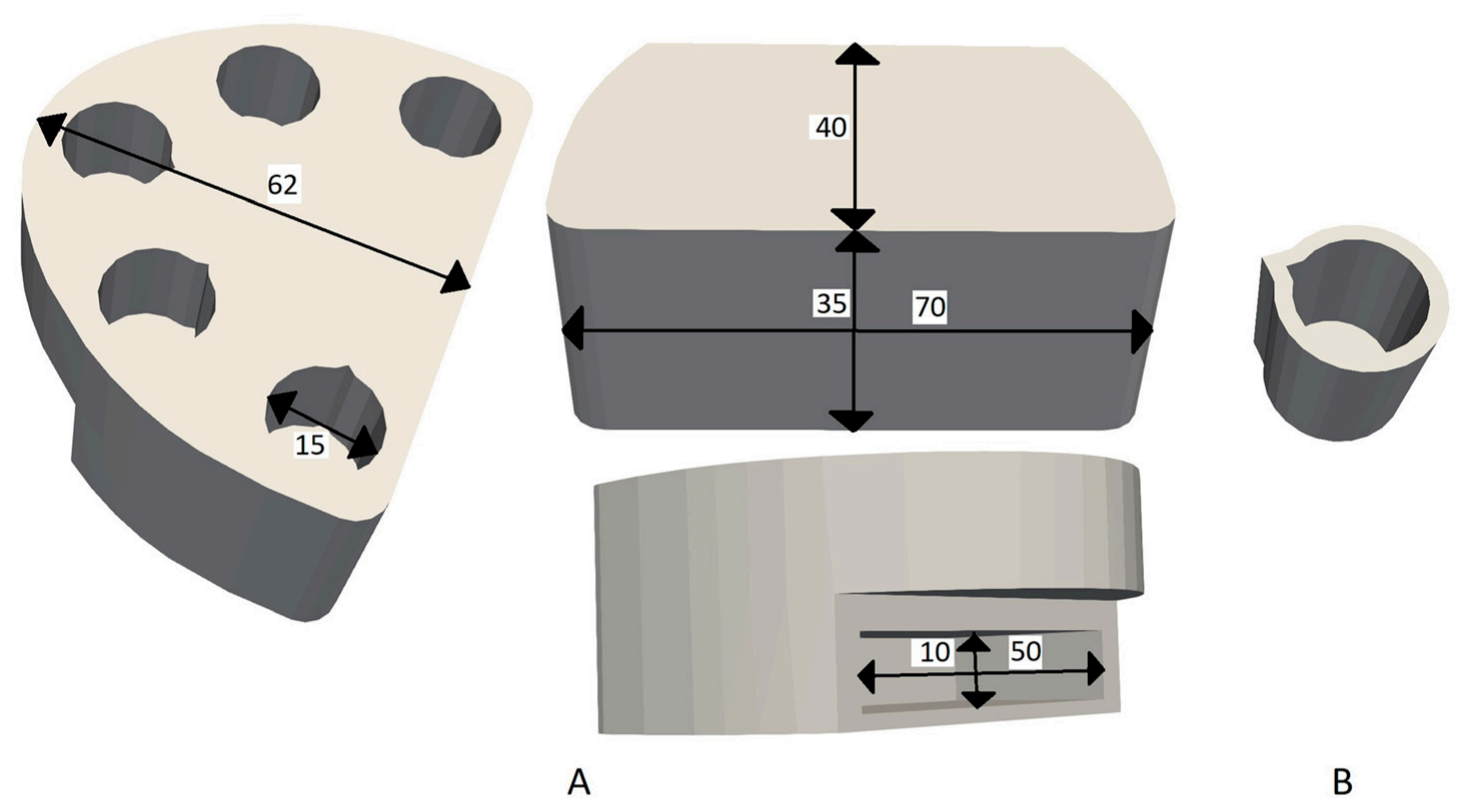
Three-dimensional-printed holder designed for reuse (**A**); Tooth support (**B**).

Based on this prototype, a definitive 3D-printed holder was digitally designed using Abaqus software (version 2023, Dassault Systèmes Simulia Corp., Providence, RI, USA). The final design (Figure 1) consists of a main structural base and five independent, interchangeable tooth supports. The overall geometry replicates the morphology of a maxillary arch, incorporating five anatomically positioned receptacles within the base to accommodate the supports. Each receptacle includes internal indentations that correspond to matching projections on the tooth supports, ensuring a precise and reproducible fit. This interlocking mechanism restricts positioning to a single orientation, thereby promoting consistent spatial alignment across repeated CBCT acquisitions and minimizing positional variability.

The modular design is intended to allow reuse of the main structure while providing flexibility in the number of tooth supports, depending on experimental requirements. Additionally, a dedicated slot is incorporated into the base to enable customized attachment and stable positioning on the CBCT headrest.

**Figure 2:**
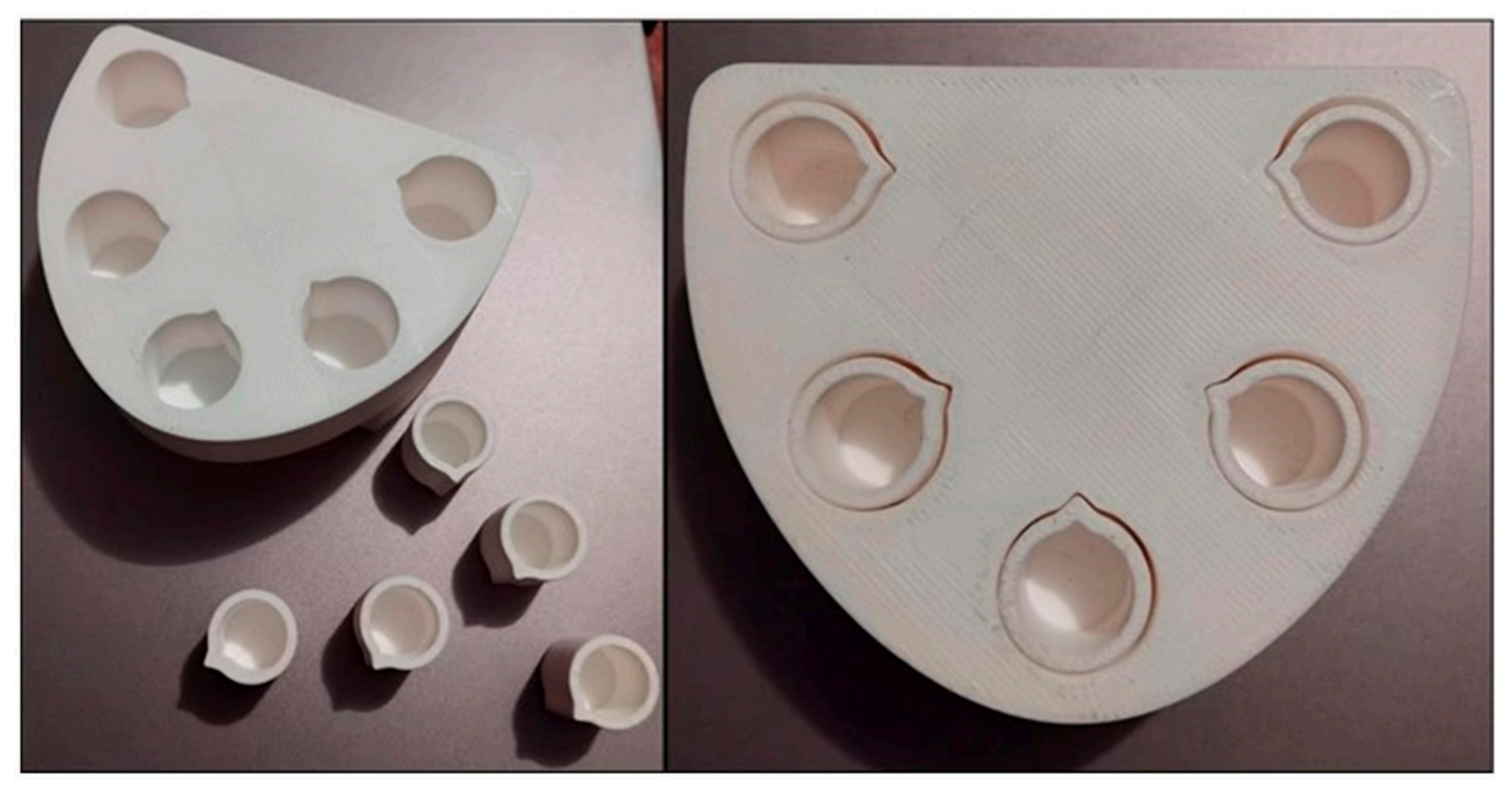
Three-dimensional-printed holder designed for reuse and five tooth supports.

The 3D-printed holder and tooth supports were fabricated at the CDRSP facilities using fused filament fabrication (FFF) technology with a Bambu Lab X1E printer (Shenzhen, China) and polylactic acid (PLA) filament. Printing was performed using a 0.4 mm nozzle, a layer thickness of 0.2 mm, 100% infill, and a nozzle temperature of approximately 220 °C. The holder was printed in an upright orientation, with its flat inferior surface in contact with the build platform and the build direction extending vertically along the height of the device. In this orientation, the openings for the interchangeable tooth supports were positioned approximately perpendicular to the build platform. The tooth supports were printed separately, with their bases in direct contact with the build platform (Figure 2).

The material used for the base and tooth supports was visible on the CBCT images. Image noise, artifacts, and image quality were not formally evaluated because no predefined qualitative or quantitative image-quality assessment was included in the study protocol. Computer-aided design files for both components are available as Supplementary Materials (File S1 (Holder) and File S2 (Tooth Support)).

A conventional holder was fabricated using a rubber container (Mestra^®^, MESTRA, Biscaye, Spain) and molded with a high-performance alginate material (Hydrogum 5, Zhermack SpA, Badia Polesine, Italy).

An initial pool of fourteen permanent teeth, extracted for clinical reasons unrelated to this study, was collected and stored in distilled water until use. Radiographs were obtained in mesiodistal and buccolingual projections to confirm eligibility. Inclusion criteria comprised fully formed apices, absence of root fractures, no evidence of internal or external resorption, absence of carious lesions in the region of interest, and no prior endodontic treatment. From this pool, five teeth were selected: two mandibular incisors, two mandibular premolars, and one maxillary premolar. The study included five teeth, two operators, two acquisition time points, and eight CBCT acquisitions.

**Figure 3:**
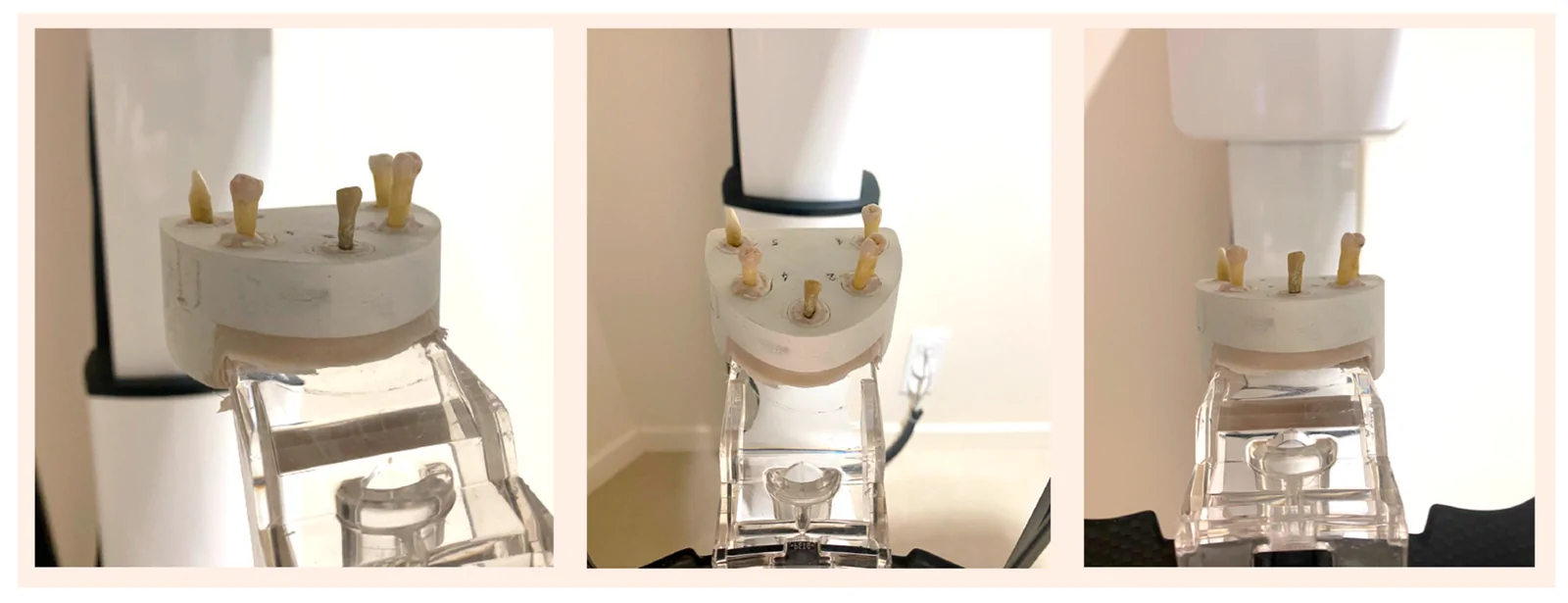
Three-dimensional-printed holder designed for reuse adapted to the CBCT headrest using high-performance alginate.

**Figure 4:**
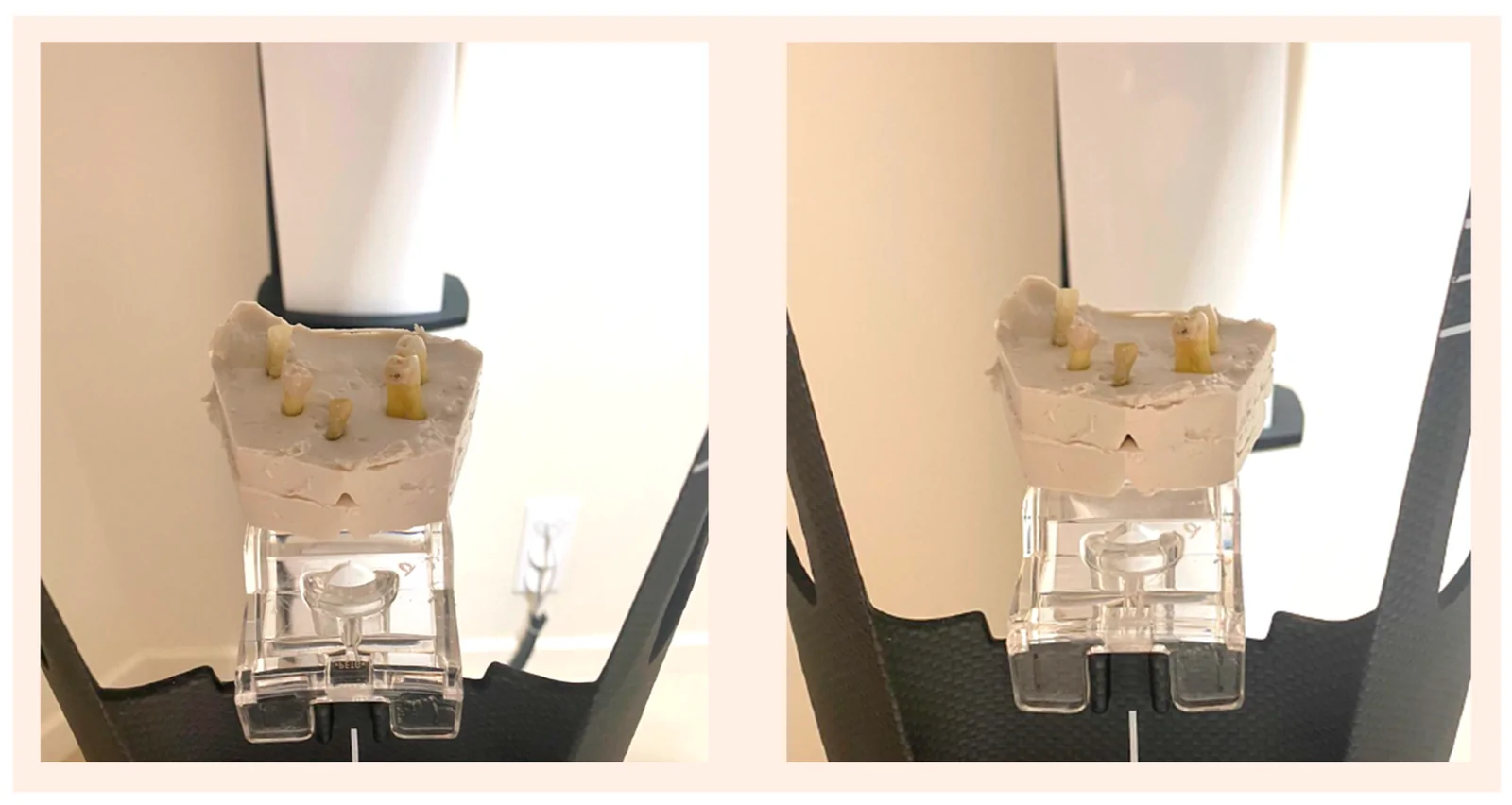
Conventional alginate-based holder positioned on the CBCT headrest.

Teeth were fixed to the tooth supports of the 3D-printed holder using high-performance alginate (Hydrogum 5, Zhermack SpA, Italy) to ensure comparability with the conventional holder and to provide stable fixation during scanning. The tooth support design accommodates both shallow and deep embedding of the teeth, allowing either external mounting for easier CBCT segmentation or deeper insertion to simulate periradicular tissue and enhance anatomical realism. In the present study, the external configuration was selected to facilitate segmentation during image analysis. To ensure precise adaptation to the CBCT head support (Carestream CS 8200 3D), the 3D-printed holder was customized using the same alginate material (Figure 3). The conventional holder was positioned on the head support with the aim of maintaining a consistent placement across all acquisitions (Figure 4). Accordingly, the experimental comparison involved the complete functional configuration of each positioning system, including its geometry, fixation mechanism, insertion constraints, and mode of adaptation to the CBCT headrest.

Two operators participated in the study: a dentist with seven years of clinical experience and a dental clinic receptionist with no clinical experience. Both operators received standardized instructions from the first author (S.T.) regarding the use of both positioning systems. The receptionist was intentionally selected as a non-expert operator with no clinical, radiological, or dental research experience, thereby providing a clear contrast in prior experience. This comparison was intended to explore whether the positioning devices could be used after standardized instructions by an individual unfamiliar with dental imaging procedures. Each operator independently positioned the teeth within the 3D-printed holder and the conventional holder and subsequently placed the devices on the CBCT unit. All scans were acquired by the same operator (S.T.). The entire procedure was repeated after 5 days, resulting in a total of eight CBCT acquisitions. The same alginate fixation was maintained and reused for the second acquisition and was not replaced between the baseline and 5-day assessments. During the 5-day interval, both positioning systems were stored in a hermetically sealed bag at room temperature (23 °C/73 °F), following the alginate manufacturer’s instructions.

All CBCT acquisitions were performed using a Carestream CS 8200 3D Access system (Carestream Dental LLC, Atlanta, GA, USA) with a field of view of 8 × 9 cm and an isotropic voxel size of 150 μm. The acquisition parameters were 90 kV, 8.0 mA, and an exposure time of 10.0 s. Images were reconstructed using the manufacturer’s Standard (STD) reconstruction mode. The reported dose-area product was 1257 mGy·cm^2^. The holder was positioned centrally within the selected field of view, with the tooth-bearing surface oriented superiorly, and was adapted to the CBCT headrest using high-performance alginate. The same holder orientation and acquisition protocol were maintained for all scans. The CBCT unit was legally authorized for clinical use and underwent the routine calibration and maintenance procedures provided by the manufacturer. No additional study-specific calibration or quality-control procedure was performed immediately before image acquisition. The equipment was used under the same operational conditions routinely applied for patient imaging.

All CBCT datasets were exported in DICOM format and imported separately into 3D Slicer (version 5.6.2; Slicer Community, Boston, MA, USA). Each root canal system was segmented independently from its corresponding original DICOM dataset. The same segmentation thresholds, tools, and processing parameters were applied consistently to all datasets. No segmentation mask, label map, or three-dimensional model was copied, propagated, transformed, or reused between datasets. The segmented canal systems were used to obtain voxel count, volume (mm^3^), and surface area (mm^2^).

Positional discrepancies were assessed in 3D Slicer (version 5.6.2, Slicer Community) by measuring the linear Euclidean distance between the apical endpoints of corresponding segmented root canals in repeated CBCT acquisitions. Each root canal system was segmented independently in each dataset, and the apical endpoint was identified on the respective 3D reconstruction. No rigid registration, image registration, mesh alignment, manual pre-alignment, or other spatial alignment procedure was performed before measurement. No anatomical landmarks, holder structures, external markers, or canal surfaces were used as registration references, and no registration algorithm was applied. Consequently, the measured distance represented the overall positional discrepancy between acquisitions in the native coordinate space of the datasets and could include both translational and rotational components. Measurements were obtained for intra-operator comparisons (dentist baseline versus 5 days [D0–D5]; receptionist baseline versus 5 days [R0–R5]) and inter-operator comparisons (D0 versus R0; D5 versus R5), for both positioning systems. Although 3D Slicer calculates distances from continuous spatial coordinates and reports decimal values, these numerical values were not interpreted as demonstrating measurement accuracy below the isotropic voxel size of 0.15 mm. No registration procedure was performed; therefore, no sub-voxel registration method or registration-related error was involved. All segmentation and measurement procedures were performed by the same operator (S.T.).

### Statistical Analysis

Statistical analyses were performed using IBM SPSS Statistics version 30.0 (IBM Corp., Armonk, NY, USA). The tooth was considered the experimental unit. Because the same five teeth were evaluated using both positioning systems, comparisons between the 3D-printed and conventional holders were treated as paired observations. For each acquisition comparison (D0 vs. D5, R0 vs. R5, D0 vs. R0, and D5 vs. R5), the distribution of the paired differences was assessed using the Shapiro–Wilk test. As no significant deviation from normality was detected, paired samples *t*-tests were used to compare positional deviations between the two holders. The Holm procedure was applied to adjust for the four paired comparisons. Mean paired differences, 95% confidence intervals, and Cohen’s dz effect sizes were reported. All tests were two-sided, with a significance level of 0.05. Holm-adjusted *p* values are reported because they offer rigorous control of the type I error rate across multiple comparisons and provide greater statistical power than the Bonferroni method. No a priori power analysis was performed because the study was designed as an exploratory proof-of-concept investigation using a convenience sample of five teeth. Given the small number of experimental units, individual tooth-level positional deviations were also reported for each acquisition comparison and positioning system. Paired values obtained with the 3D-printed and conventional holders were additionally displayed graphically, with measurements from the same tooth connected to illustrate the within-tooth comparisons.

## 3. Results

The voxel count, volume, and surface-area values were identical for each canal system across all eight CBCT datasets at the numerical precision reported by 3D Slicer (Table 1 and Table S1).

The positional deviations obtained with the two positioning systems are presented in Table 2.

**Figure 5:**
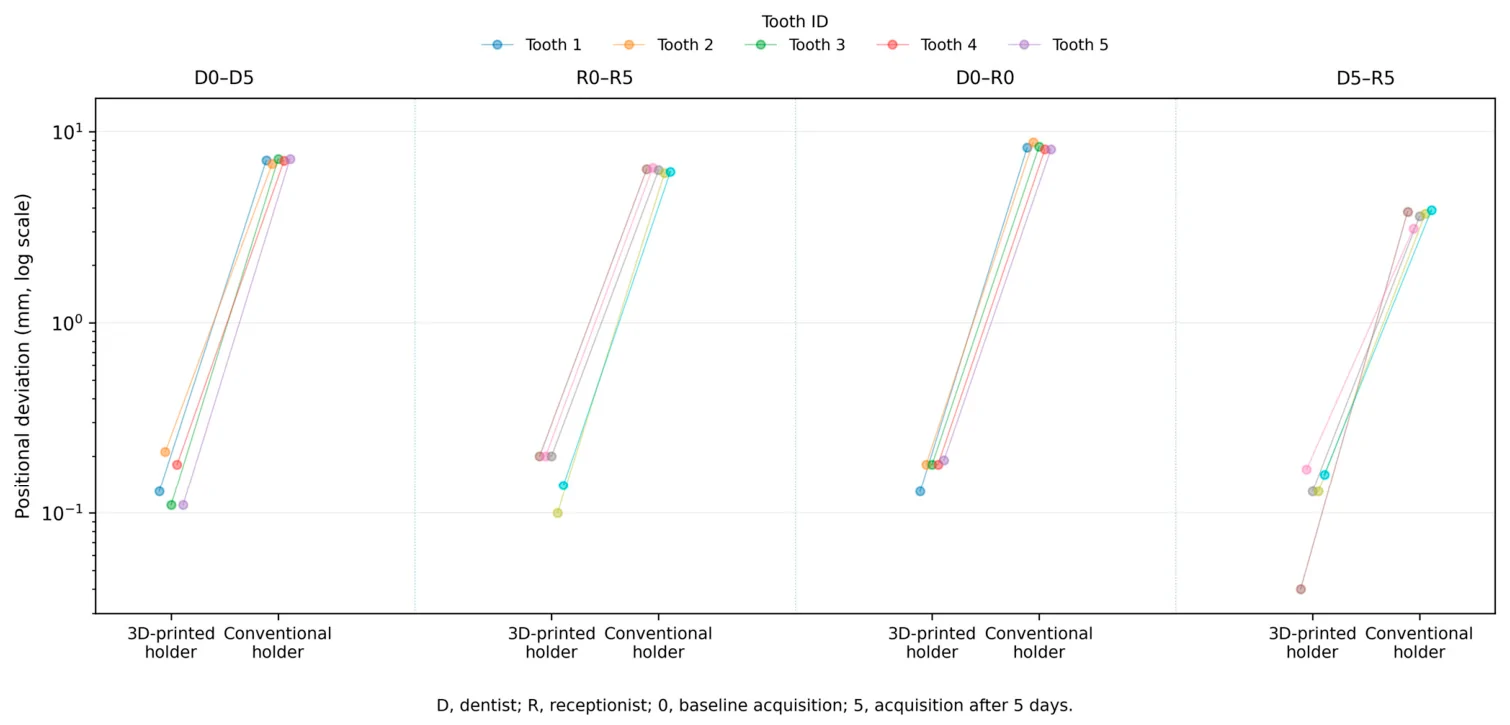
Individual paired positional deviations obtained with the 3D-printed and conventional holders across the four acquisition comparisons. Each line connects the measurements obtained from the same tooth. D, dentist; R, receptionist; 0, baseline acquisition; 5, acquisition after 5 days.

Paired analyses demonstrated lower positional deviations with the 3D-printed holder than with the conventional holder in all four acquisition comparisons. Mean paired differences ranged from 3.500 to 8.146 mm, and all Holm-adjusted comparisons were statistically significant (adjusted *p* < 0.001). The corresponding 95% confidence intervals did not include zero, and the numerical paired effect-size estimates are presented in Table 2. Mean deviations obtained with the 3D-printed holder ranged from 0.126 to 0.172 mm, whereas those obtained with the conventional holder ranged from 3.626 to 8.318 mm. Individual tooth-level positional deviations for each of the four acquisition comparisons are presented in Table S2 and Figure 5. For all five teeth and across all four comparisons, the positional deviation obtained with the 3D-printed holder was lower than the corresponding deviation obtained with the conventional holder.

**Figure 6:**
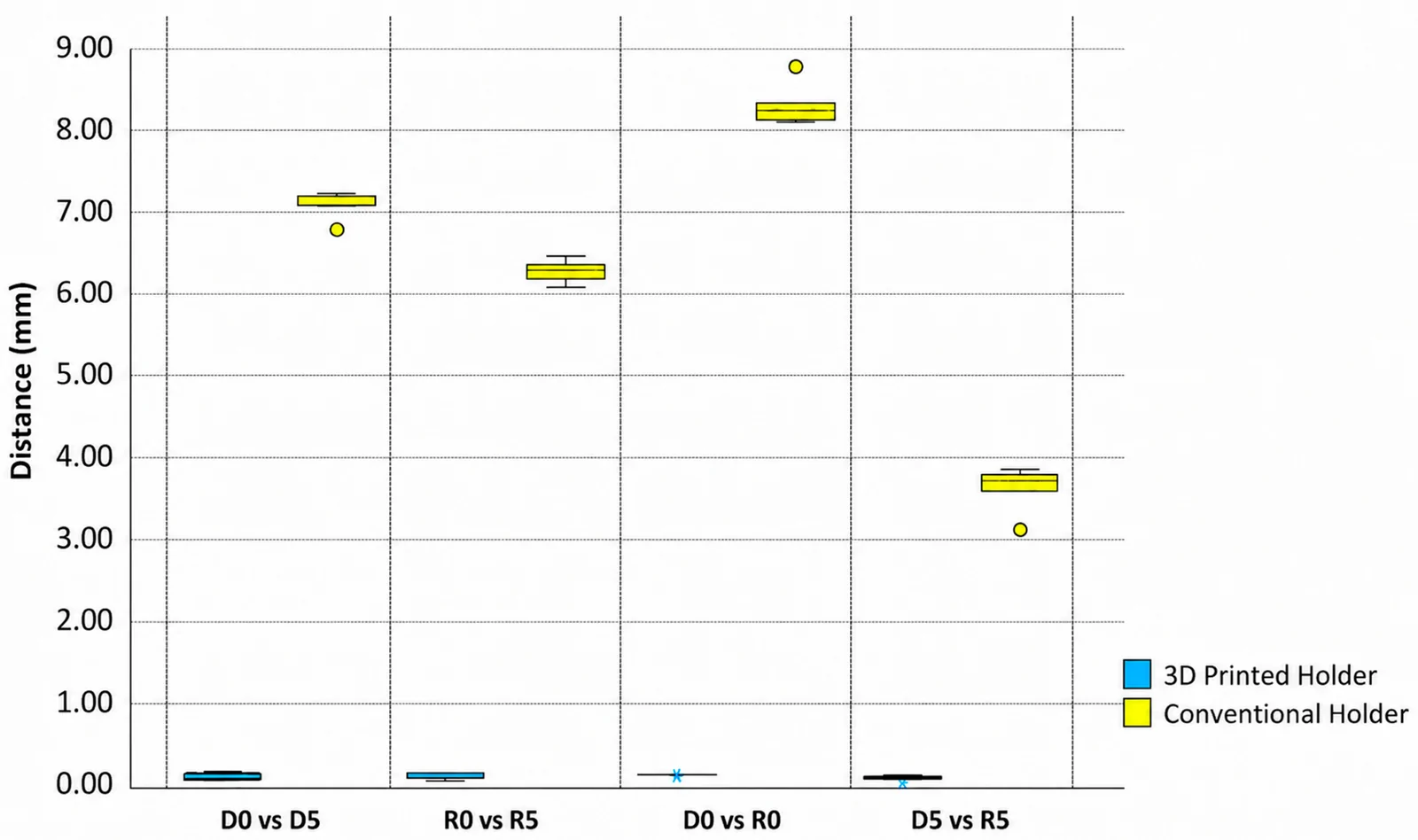
Boxplot representation of distance deviations (mm) between CBCT acquisitions using the 3D-printed and conventional holders. (D: dentist; R: receptionist; 0: baseline; 5: after 5 days). Circles indicate outliers, and asterisks (*) indicate extreme values.

Figure 6 shows the distribution of positional deviations for the two positioning methods, with a clear separation between the observed values. The 3D-printed holder presented consistently lower positional deviations, whereas the conventional holder showed substantially higher values.

**Figure 7:**
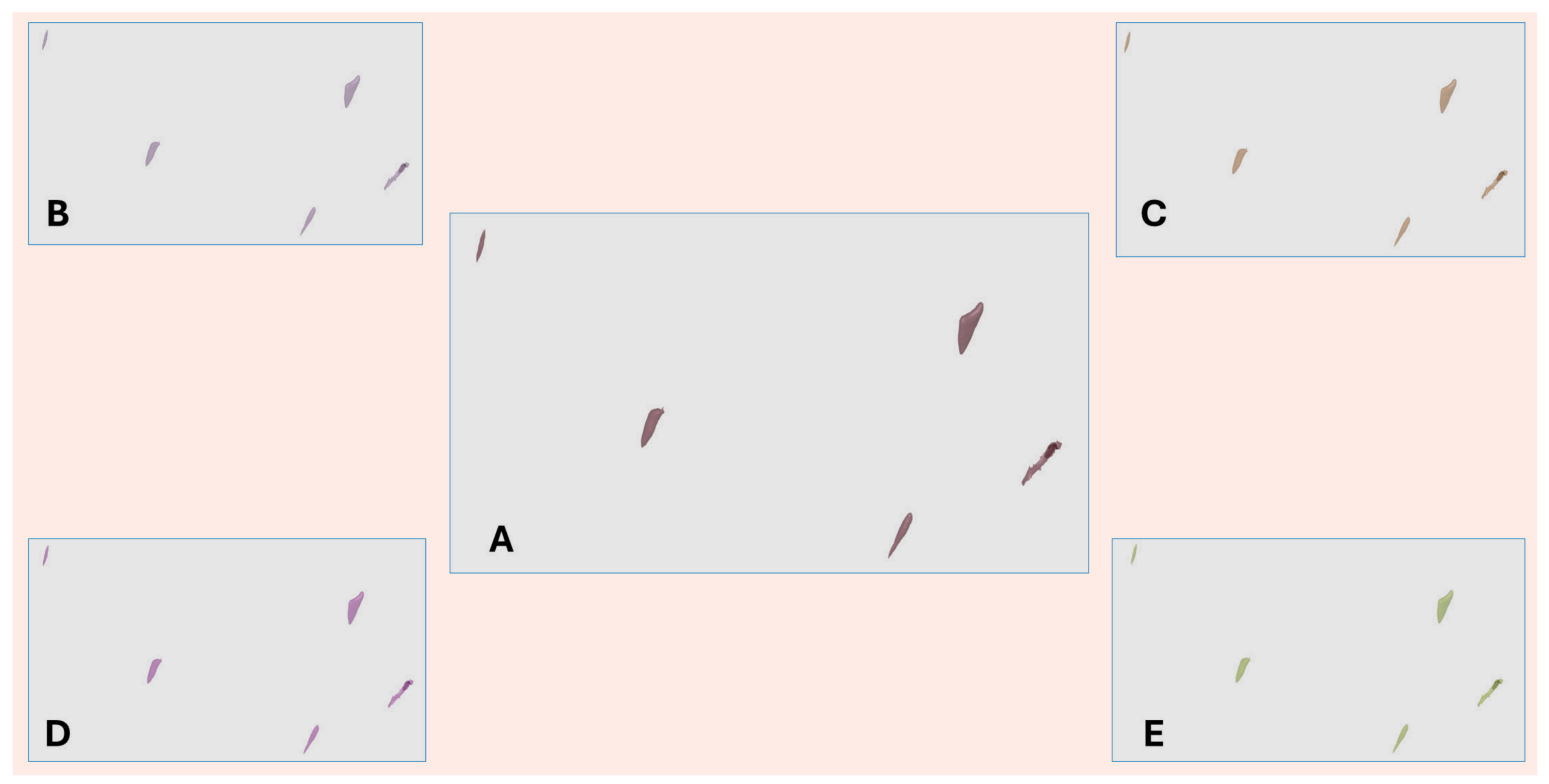
Representative combined visualization of canal systems obtained from CBCT acquisitions using the 3D-printed holder. (**A**) Combined visualization of all four acquisitions; (**B**) D0 vs. D5; (**C**) R0 vs. R5; (**D**) D0 vs. R0; (**E**) D5 vs. R5. Color coding: D0—blue; D5—orange; R0—red; R5—green. A high degree of spatial overlap is observed across all acquisitions, resulting in color blending due to minimal positional discrepancies.

**Figure 8:**
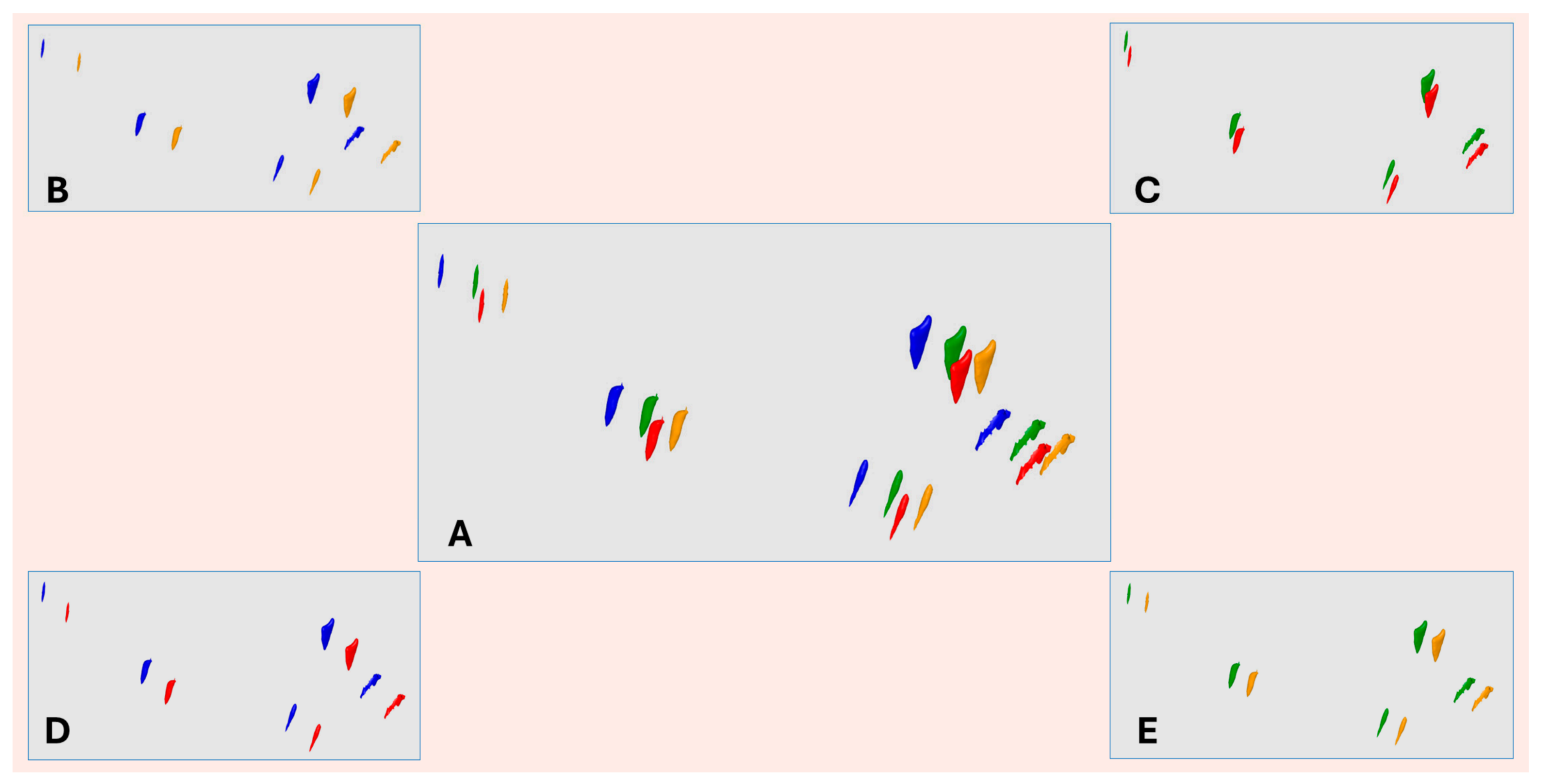
Representative combined visualization of canal systems obtained from CBCT acquisitions using the conventional holder. (**A**) Combined visualization of all four acquisitions; (**B**) D0 vs. D5; (**C**) R0 vs. R5; (**D**) D0 vs. R0; (**E**) D5 vs. R5. Color coding: D0—blue; D5—orange; R0—red; R5—green.

Representative 3D reconstructions are shown in Figure 7 and Figure 8. For the 3D-printed holder, a high degree of overlap between acquisitions was observed across all canal systems. In contrast, visible spatial discrepancies between corresponding structures were observed with the conventional holder.

## 4. Discussion

The present exploratory proof-of-concept study aimed to develop and preliminarily evaluate a modular 3D-printed positioning system designed for reuse in repeated CBCT acquisitions. The study included five teeth, two operators, two acquisition time points separated by five days, and eight CBCT datasets generated from repeated acquisitions of the same experimental units. Volumetric and surface measurements, together with the root canal voxel count, were analyzed for the five teeth across all CBCT acquisitions.

To obtain quantitative data from the root canal system, segmentation of the endodontic structures is essential. Segmentation refers to a post-processing technique that partitions an image into distinct, uniform regions. In the context of dental imaging, this process serves to delineate the pulp space from adjacent tissues such as dentin [4,25,26,27,28,29]. This technique is widely used to identify and define the shape and boundaries of anatomical structures, including contours and edges. Manual segmentation, although effective, is labor-intensive and time-consuming, particularly when assessing volumetric parameters [26,27,28,29]. To address this, automated segmentation algorithms have been developed to accelerate the process. These methods classify image elements (e.g., pixels or voxels) based on shared characteristics, distinguishing them from neighboring regions with differing properties [4,25,27,29]. When applied to sequential medical images, such as CBCT slices, segmentation enables the generation of 3D reconstructions of anatomical structures. The accuracy of morphometric analysis is highly dependent on the segmentation method employed, as different techniques may yield varying results [26,29]. One of the main challenges in automated segmentation of medical images lies in the limited contrast and spatial resolution typical of CBCT data. Consequently, no universally optimal segmentation algorithm exists [26,29]. A range of techniques—including thresholding, region growing, edge detection, and deformable models—can be applied individually or in combination, depending on the specific requirements of the dataset. An effective segmentation method is one that enhances interpretability by simplifying image content while preserving essential anatomical details [4,26,28,29]. Importantly, segmentation outcomes are influenced not only by the algorithm chosen but also by the quality of the data input. Therefore, imaging parameters used during acquisition play a critical role in determining the accuracy of the reconstructed anatomical models [4,29].

The voxel count, volume, and surface-area values remained identical across the eight CBCT datasets at the numerical precision reported by the software (Table 1 and Table S1). This finding may be related to the use of the same acquisition settings and standardized segmentation thresholds, tools, and processing parameters. Nevertheless, because each dataset was segmented only once, the exact agreement should be interpreted cautiously and should not be considered evidence of segmentation reliability or absence of measurement error.

The present findings concern positioning repeatability, defined as the ability to reproduce a similar tooth position across repeated CBCT acquisitions. They do not establish absolute positional accuracy, which would require comparison with an external reference standard or ground-truth measurement, such as an optical, mechanical, phantom-based, or higher-resolution imaging reference. Such validation was beyond the scope of this exploratory proof-of-concept study, which was intended to compare the repeated positioning performance of a newly developed modular holder designed for reuse with that of a conventional alginate-based holder. Although both systems allowed CBCT acquisition, the 3D-printed holder produced comparatively smaller positional discrepancies across operators and acquisition time points. In contrast, the conventional holder exhibited greater variability, particularly in the inter-operator comparisons. Figure 7 and Figure 8 use the same color scheme for corresponding acquisitions. Greater overlap was observed with the 3D-printed holder, resulting in visual color blending. With the conventional holder, the colors remained more clearly separated because of the greater positional discrepancies. These qualitative observations were consistent with the quantitative distance analysis. This comparison involved the complete positioning systems rather than the manufacturing materials or processes in isolation. The proposed system differed from the conventional system not only in its PLA fabrication but also in its modular geometry, interlocking mechanism, restricted insertion orientation, and adaptation to the CBCT headrest. Therefore, the differences observed cannot be attributed exclusively to PLA or to 3D printing.

From a methodological perspective, this difference has important implications. Reproducible repositioning is important for comparative CBCT analyses, particularly when evaluating morphological changes over time. Under the conditions of the present study, the 3D-printed holder exhibited lower positional discrepancies at the acquisition stage than the conventional alginate support. However, the study did not evaluate whether subsequent registration was required or quantify the time, quality, or accuracy of any registration procedure. In conventional workflows, the superimposition or alignment of datasets is often required to compensate for positioning inconsistencies. However, these processes are inherently complex, rely on mathematical algorithms that are not always transparent to the user, and may introduce additional sources of error.

The lower positional deviations observed with the 3D-printed holder suggest that this device may reduce the extent of post-processing alignment required [30]. This may represent an advantage for longitudinal and comparative CBCT studies, although its potential to shorten registration time or improve registration quality was not directly evaluated in the present study. Traditionally, scientific literature has identified three principal methods for mesh alignment: landmark-based alignment, partial area-based alignment, and entire area-based alignment. Landmark-based and partial area-based methods require the manual selection of corresponding landmarks or regions. Their performance may therefore depend on operator experience, and the procedure can be time-consuming [30,31]. In contrast, entire area-based alignments methods automatically merge two-point clouds by iteratively minimizing metric errors using a Gaussian best-fit algorithm, and do not involve operator-based decisions [31]. However, if a large defect is present, the algorithm will focus on minimizing the absolute distance between the two datasets, without considering the outcome [30], and without initial landmark-based registration, the algorithm is unlikely to yield reliable results. Several factors, including the chosen matching algorithm, the method used to define the region of interest, and the quality of the initial alignment, influence the accuracy of the final alignment outcome [32].

The present study used the Carestream CS 8200 3D system. However, headrest geometry and field-of-view dimensions vary among CBCT devices. FOVs can be classified by height as small (<100 mm), medium (100–150 mm), or large (150–200 mm) [33]. A slot was incorporated into the holder design to permit customization to the head support and field-of-view requirements of different CBCT systems. In the present study, however, the holder was evaluated only with the Carestream CS 8200 3D system and a single acquisition protocol. Therefore, compatibility with other CBCT devices and acquisition geometries remains to be demonstrated. For the system evaluated, customization was achieved using high-performance alginate, which allowed removable fixation to the CBCT headrest. This approach enabled the 3D-printed holder to be securely positioned within the CBCT FOV, ensuring consistent placement across multiple imaging scans.

The 3D-printed holder was fabricated using FFF technology and PLA. FFF is a material-extrusion additive manufacturing technique in which components are produced layer by layer [34]. Its main advantages include simplicity, accessibility, and relatively low equipment and material costs [34,35]. PLA is a biodegradable polymer derived from renewable biological sources and is often described as a more sustainable alternative to conventional petroleum-based polymers. Previous studies have reported that PLA may tolerate repeated sterilization procedures [34,35]. However, the effect of repeated cleaning, disinfection, or sterilization cycles on the dimensional stability and positioning performance of the present holder was not evaluated.

The descriptions of holders used in in vitro CBCT scanning studies are inconsistent. Some studies do not report on the positioning device used [13,14], whereas others describe a wide variety of holder designs and materials. Reported positioning approaches include silicone impression materials [11,15,22], rubber-based holders [17,23], dry human bone [10,16,18,20,21], animal bone [24], fabricated edentulous mandible [19], gypsum [9] or polymethyl methacrylate molds [8], modeling wax [12] and arch-shaped resin holders [7]. However, these studies do not specify how these holders are secured within the patient head support system, raising concerns about stability in CBCT scans.

Aflaki et al. (2020) [7] and Khademi et al. (2022) [11] designed a holder shaped like a dental arch to optimize CBCT scan acquisition and ensure consistent positioning. In Aflaki et al. (2020) [7], the teeth were mounted in auto-polymerizing acrylic resin, whereas Khademi et al. (2022) [11] used silicone putty impression material. However, to accommodate all the teeth in their studies, they had to create four and nine separate holders, respectively. In contrast, the proposed 3D-printed holder incorporates interchangeable tooth supports within a single modular unit. This design reduces the need to manufacture separate holders for each specimen and may improve standardization in repeated CBCT acquisitions. The choice of fixation material is also relevant to the performance of the holder designed for reuse. In the present study, the same alginate fixation was reused at baseline and after five days to maintain comparability between acquisitions and between the two positioning systems. The selected alginate has a manufacturer-reported dimensional stability of up to five days and was used to stabilize both the teeth and the positioning devices. For studies extending beyond this period, a material with greater long-term dimensional stability, such as polyvinyl siloxane, may be more appropriate.

The principal limitation of the present proof-of-concept study is the small experimental sample. Only five teeth, two operators, two acquisition time points, and eight repeated CBCT acquisitions were included. The five teeth constituted the experimental units, whereas the eight datasets resulted from repeated acquisitions of those same teeth. No a priori sample-size calculation was performed, as this study was designed as an exploratory proof-of-concept investigation. The small number of experimental units limits the precision of the statistical estimates and the assessment of distributional assumptions. Therefore, *p* values should be interpreted cautiously and considered together with the paired effect sizes and confidence intervals. Although repeated acquisitions allowed an assessment of within-sample positioning repeatability, they did not increase biological diversity or compensate for the limited number and range of tooth types included. Consequently, the findings should not be interpreted as comprehensive validation of the device or generalized to all tooth types, operators, CBCT systems, acquisition protocols, or longitudinal research settings. In particular, the findings obtained with a single non-expert operator cannot be generalized to other inexperienced users. They should be interpreted only as indicating the possibility that a non-expert operator may use the device under the specific conditions tested after receiving standardized instructions. No independent mechanical calibration or external dimensional verification of the positioning system was performed. In addition, potential order effects, repeated manipulation of the positioning systems, operator fatigue, and dimensional changes in the reused alginate were not formally assessed. Although the alginate was stored according to the manufacturer’s instructions, its dimensional stability was not independently verified during the 5-day study period. Future studies should include larger and more heterogeneous dental samples, additional operators, multiple CBCT systems, different acquisition settings, and actual pre- and post-intervention designs.

Another limitation of the present study is the use of a single software package and a single operator for both root canal segmentation and apical landmark identification. Although each CBCT dataset was segmented independently and no segmentation mask, label map, or three-dimensional model was reused across acquisitions, each dataset was segmented and measured only once. Consequently, intra-observer segmentation repeatability, landmark-identification repeatability, and measurement error were not formally assessed. CBCT-based root canal segmentation may also be influenced by image contrast, voxel size, artifacts, and threshold selection. In addition, image quality and holder-related artifacts were not formally assessed. No predefined qualitative or quantitative analysis of image noise, artifact magnitude, or image quality was performed. Importantly, the positional deviations observed with the 3D-printed holder (0.13–0.17 mm) were close to the isotropic voxel size used in this study (0.15 mm). These values should therefore be interpreted within the uncertainty associated with CBCT spatial resolution, segmentation, and manual identification of the apical landmark. Although the software reports distances using continuous spatial coordinates and decimal values, these outputs should not be interpreted as evidence of true sub-voxel measurement accuracy. Accordingly, values are reported to three decimal places to preserve the numerical output and avoid excessive rounding, rather than to imply a corresponding level of measurement accuracy. The measured distance between acquisitions may reflect at least three components: actual variation in tooth position, variability introduced during independent segmentation, and variability in apical landmark identification. Therefore, the observed distance should not be interpreted as a purely mechanical measure of tooth displacement. Future studies should include repeated segmentation and measurement sessions, multiple observers, and appropriate estimates of intra- and inter-observer reliability and measurement error.

The modular positioning system was designed for reuse, as only the interchangeable tooth supports need to be adapted to different experimental configurations. However, repeated-use performance was not validated in the present study. This design may therefore offer a practical advantage over single-use or case-specific holders and may contribute to more resource-efficient research workflows. However, printing time, filament consumption, production and fixation-material costs, the maximum number of reuse cycles, dimensional changes after repeated use, the effects of cleaning, disinfection, and sterilization, and the environmental impact were not evaluated in the present study.

Nonetheless, this exploratory proof-of-concept study suggests that the proposed 3D-printed holder designed for reuse may reduce tooth-positioning deviations during repeated CBCT acquisitions under controlled in vitro conditions. The extreme Cohen’s dz estimates reflect the substantial paired differences and the low within-pair variability recorded in this small sample. Given the inclusion of only five experimental units, these estimates should be interpreted cautiously and should not be considered precise or broadly generalizable estimates of effect magnitude. These observations warrant further evaluation but should not be interpreted as establishing the system’s performance across all CBCT research applications. The proposed holder is based on an accessible manufacturing approach and incorporates modular components designed for reuse, which may offer practical advantages for research settings with limited resources, including undergraduate research initiatives.

## 5. Conclusions

This exploratory proof-of-concept study, based on a convenience sample of five extracted teeth, suggests that, under the specific experimental conditions evaluated, the complete 3D-printed holder designed for reuse may reduce positional deviations and improve tooth-positioning repeatability compared with the conventional alginate-based positioning system used in this study. However, repeated-use performance, including dimensional stability over multiple cycles and the effects of cleaning, disinfection, and sterilization, was not evaluated. These findings should not be interpreted as isolating the effect of PLA or of the 3D-printing process itself. The study did not assess absolute positional accuracy relative to an external reference standard. Further studies with larger and more diverse samples and independent reference methods are required before broader conclusions can be drawn.

## Figures and Tables

**Table 1:** Voxel count, volume (mm^3^), and surface area (mm^2^) measurements for each canal system, as determined from each CBCT scan.

Canal System	Voxel Count	Volume	Area
1	829	2.80	13.51
2	2681	10.47	34.69
3	1972	6.66	31.08
4	2470	8.33	25.58
5	1040	3.51	14.32

**Table 2:** Paired comparison of positional deviations between the 3D-printed and conventional holders. Differences were calculated as conventional holder minus 3D-printed holder. *p* values were obtained using paired-samples *t*-tests and adjusted for four comparisons using the Holm procedure.

	3D-Printed Holder Mean ± SD (mm)	Conventional Holder Mean ± SD (mm)	Mean Paired Difference (mm)	95% CI	Adjusted *p*	Cohen’s dz
D0 vs. D5	0.148 ± 0.045	7.064 ± 0.167	6.916	6.658–7.174	<0.001	33.26
R0 vs. R5	0.168 ± 0.046	6.274 ± 0.153	6.106	5.968–6.244	<0.001	54.88
D0 vs. R0	0.172 ± 0.024	8.318 ± 0.294	8.146	7.780–8.512	<0.001	27.64
D5 vs. R5	0.126 ± 0.051	3.626 ± 0.307	3.500	3.085–3.915	<0.001	10.48

## Data Availability

The data that support the finding of this study are available from the corresponding author upon reasonable request.

## Supplementary Materials

The following supporting information can be downloaded at: https://www.mdpi.com/article/10.3390/jfb17080385/s1, File S1. Computer-aided design file of the 3D-printed holder in STL format; File S2. Computer-aided design files of the tooth support in STL format; Table S1. Individual voxel count, volume (mm^3^), and surface area (m^2^) measurements for each canal system across the eight independently processed CBCT datasets; Table S2. Individual positional deviations recorded for each tooth with the 3D-printed and conventional holders across the four acquisition comparisons.

## Abbreviations

The following abbreviations are used in this manuscript:

Micro-CT	Micro-computed tomography
3D	Three-dimensional
CBCT	Cone beam computed tomography
DICOM	Digital Imaging and Communications in Medicine
FFF	Fused filament fabrication
PLA	Polylactic acid filament
FOV	Field of view
