# Supplementary material for: CBCT in Dental Research: Is Complexity Necessary? An Exploratory Proof-of-Concept Study
Source: J Funct Biomater. 2026 Aug 4;17(8):385. doi: 10.3390/jfb17080385 (PMC13514125; doi:10.3390/jfb17080385)
Supplement: Supplementary file 1 [file jfb-17-00385-s001.zip › Table S2. Individual positional deviations.pdf]

Table S2. Individual positional deviations recorded for each tooth with the 3D-printed and conventional holders across the four acquisition comparisons.

Tooth ID	Tooth type	Compariso n	Acquisition 1 (operator/time)	Acquisition 2 (operator/time)	3D-printed holder (mm)	Conventional holder (mm)	Paired difference (mm)
Tooth 1	Mandibular incisor	D0–D5	Dentist, baseline	Dentist, 5 days	0.13	7.08	6.95
Tooth 1	Mandibular incisor	R0–R5	Receptionist, baseline	Receptionist, 5 days	0.20	6.36	6.16
Tooth 1	Mandibular incisor	D0–R0	Dentist, baseline	Receptionist, baseline	0.13	8.25	8.12
Tooth 1	Mandibular incisor	D5–R5	Dentist, 5 days	Receptionist, 5 days	0.04	3.81	3.77
Tooth 2	Mandibular incisor	D0–D5	Dentist, baseline	Dentist, 5 days	0.21	6.79	6.58
Tooth 2	Mandibular incisor	R0–R5	Receptionist, baseline	Receptionist, 5 days	0.20	6.46	6.26
Tooth 2	Mandibular incisor	D0–R0	Dentist, baseline	Receptionist, baseline	0.18	8.81	8.63
Tooth 2	Mandibular incisor	D5–R5	Dentist, 5 days	Receptionist, 5 days	0.17	3.11	2.94
Tooth 3	Mandibular premolar	D0–D5	Dentist, baseline	Dentist, 5 days	0.11	7.18	7.07
Tooth 3	Mandibular premolar	R0–R5	Receptionist, baseline	Receptionist, 5 days	0.20	6.30	6.10
Tooth 3	Mandibular premolar	D0–R0	Dentist, baseline	Receptionist, baseline	0.18	8.34	8.16
Tooth 3	Mandibular premolar	D5–R5	Dentist, 5 days	Receptionist, 5 days	0.13	3.60	3.47
Tooth 4	Mandibular premolar	D0–D5	Dentist, baseline	Dentist, 5 days	0.18	7.06	6.88
Tooth 4	Mandibular premolar	R0–R5	Receptionist, baseline	Receptionist, 5 days	0.10	6.07	5.97
Tooth 4	Mandibular premolar	D0–R0	Dentist, baseline	Receptionist, baseline	0.18	8.11	7.93
Tooth 4	Mandibular premolar	D5–R5	Dentist, 5 days	Receptionist, 5 days	0.13	3.73	3.60
Tooth 5	Maxillary premolar	D0–D5	Dentist, baseline	Dentist, 5 days	0.11	7.21	7.10
Tooth 5	Maxillary premolar	R0–R5	Receptionist, baseline	Receptionist, 5 days	0.14	6.18	6.04
Tooth 5	Maxillary premolar	D0–R0	Dentist, baseline	Receptionist, baseline	0.19	8.08	7.89
Tooth 5	Maxillary premolar	D5–R5	Dentist, 5 days	Receptionist, 5 days	0.16	3.88	3.72

Abbreviations: D, dentist; R, receptionist; 0, baseline acquisition; 5, acquisition after 5 days. Paired difference was calculated as conventional holder minus 3D-printed holder.
