# Supplementary material for: CBCT in Dental Research: Is Complexity Necessary? An Exploratory Proof-of-Concept Study
Source: J Funct Biomater. 2026 Aug 4;17(8):385. doi: 10.3390/jfb17080385 (PMC13514125; doi:10.3390/jfb17080385)
Supplement: Supplementary file 1 [file jfb-17-00385-s001.zip › Table S1. Individual morphometric measurements.pdf]

Table S1. Individual voxel count, volume (mm³), and surface area (mm²) measurements for each canal system across the eight independently processed CBCT datasets.

Canal System	Positioning System	Acquisition	Voxel Count	Volume (mm³)	Surface Area (mm²)
1	3D-printed holder	D0	829	2.80	13.51
1	3D-printed holder	D5	829	2.80	13.51
1	3D-printed holder	R0	829	2.80	13.51
1	3D-printed holder	R5	829	2.80	13.51
1	Conventional holder	D0	829	2.80	13.51
1	Conventional holder	D5	829	2.80	13.51
1	Conventional holder	R0	829	2.80	13.51
1	Conventional holder	R5	829	2.80	13.51
2	3D-printed holder	D0	2681	10.47	34.69
2	3D-printed holder	D5	2681	10.47	34.69
2	3D-printed holder	R0	2681	10.47	34.69
2	3D-printed holder	R5	2681	10.47	34.69
2	Conventional holder	D0	2681	10.47	34.69
2	Conventional holder	D5	2681	10.47	34.69
2	Conventional holder	R0	2681	10.47	34.69
2	Conventional holder	R5	2681	10.47	34.69
3	3D-printed holder	D0	1972	6.66	31.08
3	3D-printed holder	D5	1972	6.66	31.08
3	3D-printed holder	R0	1972	6.66	31.08
3	3D-printed holder	R5	1972	6.66	31.08
3	Conventional holder	D0	1972	6.66	31.08
3	Conventional holder	D5	1972	6.66	31.08
3	Conventional holder	R0	1972	6.66	31.08
3	Conventional holder	R5	1972	6.66	31.08
4	3D-printed holder	D0	2470	8.33	25.58
4	3D-printed holder	D5	2470	8.33	25.58
4	3D-printed holder	R0	2470	8.33	25.58
4	3D-printed holder	R5	2470	8.33	25.58
4	Conventional holder	D0	2470	8.33	25.58
4	Conventional holder	D5	2470	8.33	25.58
4	Conventional holder	R0	2470	8.33	25.58
4	Conventional holder	R5	2470	8.33	25.58
5	3D-printed holder	D0	1040	3.51	14.32
5	3D-printed holder	D5	1040	3.51	14.32
5	3D-printed holder	R0	1040	3.51	14.32
5	3D-printed holder	R5	1040	3.51	14.32
5	Conventional holder	D0	1040	3.51	14.32
5	Conventional holder	D5	1040	3.51	14.32
5	Conventional holder	R0	1040	3.51	14.32
5	Conventional holder	R5	1040	3.51	14.32

Abbreviations: D, dentist; R, receptionist; 0, baseline acquisition; 5, acquisition after 5 days.
